# Predicting successful ageing among older adults seems possible even as far as two decades ahead

**DOI:** 10.1186/s12877-024-05109-8

**Published:** 2024-06-01

**Authors:** Anna Viljanen, Marika Salminen, Kerttu Irjala, Minna Löppönen, Hannele Tuori, Tero Vahlberg, Matti Viitanen, Laura Viikari

**Affiliations:** 1 The Wellbeing Services County of Southwest Finland, Turku University Hospital, Domain of General Practice and Rehabilitation, Turku, Finland; 2 Faculty of Medicine, Department of Clinical Medicine, Unit of Geriatric Medicine, University of Turku and Turku University Hospital, FI-20014, Turku, 20700 Finland; 3 The Wellbeing Services County of Southwest Finland, Medical Domain, Geriatric Medicine, Turku University Hospital, Turku, Finland; 4 Faculty of Medicine, Department of Clinical Medicine, Unit of General Practice, University of Turku and Turku University Hospital, Turku, 20014 Finland; 5 Faculty of Medicine, Department of Clinical Medicine, Unit of Clinical Chemistry, University of Turku and Turku University Hospital, Turku, 20521 Finland; 6 Health Economics, Department of Health and Social Management, University of Eastern Finland, Kuopio, Finland; 7 Faculty of Medicine, Department of Clinical Medicine, Unit of Biostatistics, University of Turku and Turku University Hospital, Turku, Finland; 8 Division of Clinical Geriatrics, Center for Alzheimer Research, Department of Neurobiology, Care Sciences and Society, Karolinska Institutet and Karolinska University Hospital, Huddinge, Stockholm, Sweden

**Keywords:** Feeling useful, Successful ageing, Self-rated health

## Abstract

**Background:**

Successful ageing is the term often used for depicting exceptional ageing and can be measured with multidimensional models including physical, psychological and social wellbeing. The aim of this study was to test multidimensional successful ageing models to investigate whether these models can predict successful ageing, and which individual subcomponents included in the models are most significantly associated with successful ageing.

**Methods:**

Successful ageing was defined as the ability to live at home without daily care at the age of 84 years or over. Data on the participants’ physical, psychological and social wellbeing were gathered at baseline and the follow-up period was 20 years. Four successful ageing models were constructed. Backward stepwise logistic regression analysis was used to identify the individual subcomponents of the models which best predicted successful ageing.

**Results:**

All successful ageing models were able to predict ageing successfully after the 20-year follow-up period. After the backward stepwise logistic regression analysis, three individual subcomponents of four models remained statistically significant and were included in the new model: having no heart disease, having good self-rated health and feeling useful. As a model, using only these three subcomponents, the association with successful ageing was similar to using the full models.

**Conclusions:**

Multidimensional successful ageing models were able to predict successful ageing after a 20-year follow-up period. However, according to the backward stepwise logistic regression analysis, the three subcomponents (absence of heart disease, good self-rated health and feeling useful) significantly associated with successful ageing performed as well as the multidimensional successful ageing models in predicting ageing successfully.

**Supplementary Information:**

The online version contains supplementary material available at 10.1186/s12877-024-05109-8.

## Background

Successful ageing (SA) has been defined various ways [1] with the most popular perhaps being the definition of physical, psychological and social well-being by Rowe and Kahn [2]. Since that, it has been postulated, that SA is also possible in the presence of disease and disabilities [3]. If only individuals with high levels of functioning are considered successful agers, they can be non-existent after a long follow-up period [4].

Nosraty et al. (2019) constructed four SA models that included different combinations of physical, psychological and social components and found that the model which allowed slight dependencies in functioning and a few illnesses, but not dementia or depression, was the most robust predictor of institutionalization among Finnish nonagenarians [5]. They have also investigated the association of SA according to the same models, with mortality among nonagenarians, and had similar results [6].

As Nosraty et al. (2019) point out, when examining older individuals, the probability of disease and disabilities is higher than in a younger population and therefore the definition of successful ageing by Rowe and Kahn (1997) (absence of disease and disability, maintenance of physical and cognitive functioning and active engagement in life) seems strict [2]. Young et al. (2009) suggested that the three components can compensate each other, so that successful ageing can occur even with disease and disability [3]. Obviously, not having serious illnesses or disabilities improves the probability of ageing successfully. The psychological component is of utter most importance as it reflects the outlook on one’s health when facing disease [7]. The association of self-rated health (SRH) with mortality has been confirmed in a large-scale meta-analysis [8]. Also, earlier research indicates that having social support when facing a health stressor lowers the risk of institutionalization among older people [9]. These findings thus support the use of multidimensional SA models.

We have previously described older adults (born in or before the year 1933) who at the age of 84 years or over were still able to live at home without daily informal or formal care, and defined them as successful agers. We found that these successful agers were satisfied with their lives and had a lower biological than chronological age both at baseline (in 1998‒1999 at the age of 64 years or over) and after a 20-year follow-up [10].

The purpose of this study was to test multidimensional SA models constructed by Nosraty et al. (2019) [5], in predicting successful ageing (independent living at home at the age of 84 years or over) in a younger (aged 64 years or over) population during a longer follow-up period of 20 years. Same construct of physical, psychological and social components were used with only minor modifications. Also, we aimed to investigate which individual subcomponents included in the SA models predicted successful ageing.

## Methods

### Study population

This study is part of the longitudinal Lieto study, a clinical, epidemiological study of subjects aged 64 years or older. It was carried out in Lieto, a semi-industrialized rural municipality in Southwestern Finland. All residents born in 1933 or earlier living in Lieto on February 16th of 1998 (*n* = 1596; 666 men and 930 women, 12% of the population) were invited, in a random order, to participate in the study at baseline. Of those eligible, 63 died before the baseline examination and 273 refused or did not respond. Altogether 1260 (82%) subjects participated in the baseline examination between March 1998 and September 1999, 533 men and 727 women. The baseline examination is described elsewhere [11]. Briefly, at baseline the study protocol consisted of an extensive interview on demographic and socioeconomic factors and health behavior, numerous laboratory tests, and a clinical examination including a comprehensive survey of the participants’ medical records. The baseline examination was carried out in the Lieto Health Care Center or at the participant’s home.

For re-examination that took place between September and November of 2018, we invited the original Lieto study participants still living at home in the municipality of Lieto in June of 2018 (*n* = 221). Before the re-examination, three were institutionalized and five deceased. Seventy-five subjects did not participate, leaving 138 participants for the re-examination. Briefly, the re-examination took place circa 20 years after the baseline examination and included most of the original variables and a few new ones [10]. The re-examination was conducted by one study physician and one study nurse and was performed either at Lieto Health Care Center or the participants’ home and included the meticulous investigation of electronic patient records to include all the diseases acquired during the 20-year follow-up.

**Fig. 1:**
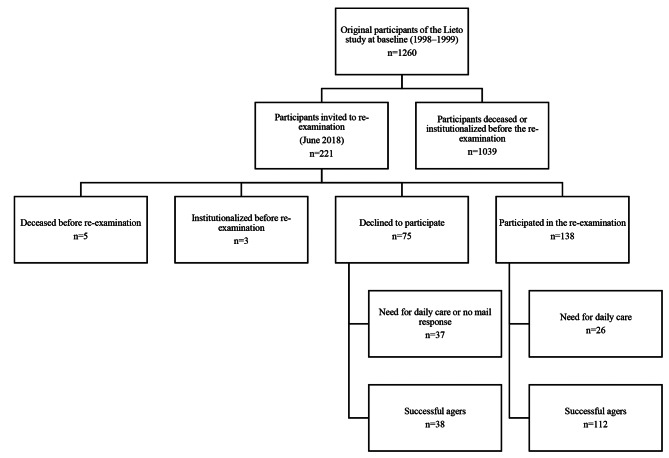
Flow chart of the study participants

Of the re-examined participants, 112 had no need for daily informal or formal care at time of re-examination and were considered successful agers [10]. Also, 38 of the non-participants responded by mail and had no need for daily informal or formal care and thus were considered successful agers and their data are used in part of the analyses. Flow chart for the study is shown in Fig. 1.

The characteristics of the re-examined successful agers (*n* = 112) at baseline and at re-examination have been reported earlier [10]. Briefly, the participants’ mean age was 67.8 years (SD 2.5, range 64‒77) at baseline and 87.6 years (SD 2.5, range 84‒96) at re-examination. In all variables analyzed, there was a statistically significant change depicting poorer physical ability and subjective health at re-examination than at baseline. Of the participants, 99% of were at least moderately satisfied with their lives and 80% described their SRH at least moderate.

### Mortality

Data from all participants who died before January 2017 were obtained from the official Finnish Cause of Death Registry using unique personal identification numbers, and from the municipality’s electronic patient record system from January 2017 to September 2018. Original participants who were deceased before the re-examination were considered as unsuccessful agers in the analyses.

### Institutionalization and need for daily informal or formal care

Institutionalization was defined as permanent entry into a long-term care facility. Daily informal care was defined as having a live-in carer providing daily care. Daily formal care was defined as having daily visits from the municipality’s home care nursing staff. Data on institutionalization from baseline to the start of the re-examination in September 2018 and data on need for daily informal or formal care at time of the re-examination were gathered from the municipality’s electronic patient record system. Participants institutionalized during the follow-up period were not invited to the re-examination. Participants institutionalized before the re-examination or needing daily informal or formal care at time of the re-examination were considered as unsuccessful agers in the analyses.

### Successful ageing models

The constructed SA-models included physical, psychological and social components each made up of different subcomponents (Additional file 1).

#### Physical component (PhC)

Diseases and functional ability were considered as the physical elements.

Diseases were considered as physician-diagnosed in contrary to the study by Nosraty et al. (2019) [5], which included only self-report data on diseases.

The following diseases were considered using the 10th revision of the International Statistical Classification of Diseases and Related Health Problems (ICD-10) [12]: heart disease (I20‒25, I48‒49), cerebral vascular diseases (I63‒64), diabetes (E11), arthritis (M05‒06, M10), Parkinson’s disease (G20), hip fracture (S72) and dementia (F00‒03, G30). Also dementia diagnosed by the Diagnostic and Statistical Manual of Mental Disorders (DSM-IV) [13] at the baseline examination was considered. In addition to these, the study physicians determined whether the participant suffered from the effects of stroke at time of the baseline examination.

Functional ability was measured as self-reported similarly as in the study by Nosraty et al. (2019) [5] by a question on the independent ability to move indoors, to get in and out of bed, to dress and undress, to walk a flight of stairs and to walk 400 m. The answer options to all of these were: “independently”, “independently but with difficulties”, “if someone helps”, and “no”. The participants were only categorized as able to do these activities if they answered either “independently” or “independently but with difficulties”. The last two of these activities were considered more demanding than the first three.

Two alternatives for PhC were constructed:

**PhC 1**: Absence of all the diseases and independence in all five activities.

**PhC2**: Absence of dementia, less than three diseases, independent in three less demanding activities (move indoors, get in and out of bed, dress and undress).

#### Psychological component (PsC)

Three elements were included: depression or depressive feelings, SRH and the feeling of leading a full life.

For depression or depressive feelings we considered previous or current diagnosis of depression in the medical records by ICD-10 [12] code of F32, diagnosis of depression by the DSM-IV [13] at time of baseline examination, and depressive feelings at time of baseline examination assessed by the Zung Depressive Scale [14] question “I feel sad and blue” with the answer options of “never or very seldom”, “sometimes”, “often” and “mostly or always”. A person was categorized as having depressive feelings if they answered “often” or “mostly or always”. The diagnosis of depression was considered as physician-diagnosed rather than self-reported as in Nosraty et al. (2019) [5].

SRH was evaluated similarly as in the study by Nosraty et al. (2019) [5] at the baseline examination by the question “How would you rate your current state of health?” with the answer options of “very good”, “good”, “average”, “poor” or “very poor”. SRH was regarded as good if answered “very good”, “good” or “average” and poor if answered “poor” or “very poor”.

In the original study, the third element included in the psychological component was the respondent’s opinion regarding the desirability of living up 100 years as a proxy measure for zest for life [5]. As our participants were younger than the nonagenarians in their study, we decided on three different measures for leading a full life, one of them also considered as a proxy measure for zest for life.

Leading a full life was evaluated by three components: satisfaction with life (gratification), hopefulness for the future (zest for life) and feeling useful (meaning). Satisfaction with life was assessed at the baseline examination by the question “How satisfied are you with your life?” with the answer options of “very satisfied”, “satisfied”, “somewhat satisfied”, “unsatisfied” and “very unsatisfied”, and was considered good if answered “very satisfied”, “satisfied” or “somewhat satisfied” and poor if answered “unsatisfied” or “very unsatisfied”. Hopefulness for the future was assessed by the question “I look hopefully into the future” with the answer options of “mostly or always”, “often”, “sometimes” or “seldom or never” and considered good if answered “mostly or always” or “often”, and poor if answered “sometimes” or “seldom or never”. Feeling useful was assessed by the question “I believe I am useful and needed” with the answer options of “mostly or always”, “often”, “sometimes” or “seldom or never”, and considered good if answered “mostly or always” or “often”, and poor if answered “sometimes” or “seldom or never”. Leading a full life was defined as being satisfied with life, having hope for the future and feeling useful.

Two alternatives for PsC were constructed:

**PsC1**: Absence of depression or depressive feelings, good SRH and leading a full life.

**PsC2**: Absence of depression or depressive feelings and good SRH.

#### Social component (SC)

Three aspects of social relationships were included: satisfaction with the relationship with a partner, with children and with friends.

In the original study, the SC was constructed as a proxy measure from the frequency of meetings with children and the frequency of telephone contacts with family members or friends [5]. However, in our study, we had available the direct information on the participants’ satisfaction with the relationships with their partners, children and friends and therefore used those in the construction of the SC.

Satisfaction with the relationship with one’s partner was assessed by the question “How satisfied are you or were you with the closeness and confidentiality of the relationship with your partner?” with the answer options of “very satisfied”, “satisfied”, “somewhat satisfied”, “somewhat unsatisfied”, “very unsatisfied” and “I have not had a partner”. Participants were considered satisfied with their relationship with their partner if they answered “very satisfied”, “satisfied”, “somewhat satisfied” or “I have not had a partner”, and unsatisfied if they answered “somewhat unsatisfied” or “very unsatisfied”.

Satisfaction with the relationship with one’s children was assessed by the question “How satisfied are you with the closeness and confidentiality of the relationship with your children?” with the answer options of “very satisfied”, “satisfied”, “somewhat satisfied”, “somewhat unsatisfied”, “very unsatisfied” and “I do not have children”. Participants were considered satisfied with their relationship with their children if they answered “very satisfied”, “satisfied”, “somewhat satisfied” or “I do not have children”, and unsatisfied if they answered “somewhat unsatisfied” or “very unsatisfied”.

Satisfaction with the relationship with one’s friends was assessed by the question “How satisfied are you with the closeness and confidentiality of the relationship with your friends?” with the answer options of “very satisfied”, “satisfied”, “somewhat satisfied”, “somewhat unsatisfied”, “very unsatisfied” and “I do not have any friends”. Participants were considered satisfied with their relationship with their friends if they answered “very satisfied”, “satisfied” or “somewhat satisfied”, and unsatisfied if they answered “somewhat unsatisfied”, “very unsatisfied” or “I do not have any friends”.

**SC**: Satisfaction in all three aspects of social relationships.

The four alternative models for SA and their components are presented in Table 1. All of the models include the same SC. SA model 1 included PhC1, PsC1 and SC, SA model 2 included PhC1, PsC2 and SC, SA model 3 included PhC2, PsC2 and SC, and SA model 4 included PhC2, PsC1 and SC.

**Table 1:** Successful ageing models and their components

Components of successful ageing	Successfull Ageing models			
	Model 1	Model 2	Model 3	Model 4
*Physical Component 1 (PhC1)*	●	●		
Absence of disease				
Independence in all activities				
*Physical Component 2 (PhC2)*			●	●
Absence of dementia				
Less than three diseases				
Independence in three less demanding activities				
*Psychological Component 1 (PsC1)*	●			●
No depression or depressive feelings				
Self-rated health good				
Leading a full life				
*Psychological Component 2 (PsC2)*		●	●	
No depression or depressive feelings				
Self-rated health good				
*Social Component (SC)*	●	●	●	●
Safisfied with social relationships				

### Outcome

The outcome of the study was successful ageing defined as the ability to live at home without daily informal or formal care at the age of 84 years or older.

### Statistical analyses

The SA models were used as independent variables in the analyses. First, unadjusted logistic regression analyses were conducted for the association of the SA models with successful ageing. Second, logistic regression analyses were adjusted for age and gender.

Third, logistic regression analyses were conducted for the individual subcomponents of the SA models and their association with successful ageing. A backward stepwise logistic regression analysis (exclusion criteria *p* ≥ 0.05) was performed to identify the individual subcomponents which on their own predicted successful ageing. Fourth, the new model including the remaining individual subcomponents was adjusted for age and gender.

Missing data on the subcomponents lead to the exclusion of the participant from the analysis involved. When analyzing the SA models 1‒4, the number of participants excluded for missing data were 67, 79, 91 and 73, respectively. In the backward stepwise logistic regression analysis, 160 participants were excluded during the backward stepwise phase when determining the statistically significant subcomponents as they had a missing value in one or more of the subcomponents. After completion of the backward stepwise phase, a new model was constructed including the statistically significant subcomponents identified in the previous phase, and all the participants who had a value on the statistically significant subcomponents were included in the analyses of the new model. In these analyses, 75 participants were excluded for missing values.

Area under the receiver operating characteristic curve (AUC) was used to measure the ability of the SA models and the new model including the remaining individual subcomponents to predict successful ageing. In general, an AUC of 0.5 suggests no discrimination, 0.7 to 0.8 is considered acceptable, 0.8 to 0.9 is considered excellent, and more than 0.9 is considered outstanding.

The results are presented with odds ratios (OR) and their 95% confidence intervals (CI). *P* values less than 0.05 were considered statistically significant. All statistical analyses were performed using SAS System for Windows, version 9.4 (SAS Institute Inc., Cary, NC, USA).

## Results

The number of participants who met the criteria of the SA models at baseline were 313, 418, 706 and 494 for models 1‒4, respectively. The number and proportion of participants who met the criteria for the physical, psychological and social components and the subcomponents at baseline are shown in Additional file 2.

The unadjusted and adjusted ORs and their 95% CIs for the association of the SA models with successful ageing (living at home at the age of 84 years or over without daily informal or formal care) are shown in Table 2. Meeting the criteria of any model was associated with successful ageing in the unadjusted, and adjusted analyses after the 20-year follow-up period. The OR for the Model 2 in the adjusted analyses was 1.97 with a *p* value of < 0.001 describing that participants meeting the criteria of Model 2 at baseline, were more likely to be successful agers after the 20-year follow-up period. The AUCs were higher when the models were adjusted with age and gender, when all of the models had an excellent AUC of over 0.8.

**Table 2:** The association of the SA models with successful ageing after the 20-year follow-up period

	Unadjusted			Adjusted^a^		
	OR (95% CI)	*P* value	AUC	OR (95% CI)	*P* value	AUC
Model 1 (PhC1 + PsC1 + SC)	3.01 (2.10‒4.32)	< 0.001	0.619	1.89 (1.29‒2.79)	0.001	0.818
Model 2 (PhC1 + PsC2 + SC)	3.00 (2.09‒4.30)	< 0.001	0.632	1.97 (1.34‒2.90)	< 0.001	0.819
Model 3 (PhC2 + PsC2 + SC)	2.80 (1.84‒4.25)	< 0.001	0.611	1.75 (1.12‒2.74)	0.014	0.813
Model 4 (PhC2 + PsC1 + SC)	2.93 (2.03‒4.21)	< 0.001	0.631	1.86 (1.27‒2.75)	0.002	0.819

OR = Odds Ratio

CI = Confidence Interval

AUC = Area Under the Curve

PhC1 = Physical Component 1: Absence of all the diseases and independence in all five activities

PhC2 = Physical Component 2: Absence of dementia, less than three diseases, independent in three less demanding activities (move indoors, get in and out of bed, dress and undress)

PsC1 = Psychological Component 1: Absence of depression or depressive feelings, good self-rated health and leading a full life

PsC2 = Psychological Component 2: Absence of depression or depressive feelings and good self-rated health

SC = Social Component: Satisfaction in all three components of social relationships

^a^Adjusted for age and gender

After the backward stepwise logistic regression analysis, three individual subcomponents of four SA models remained statistically significant and were included in the new model: physical subcomponent 1 (phc1: having no heart disease), psychological subcomponent 4 (psc4: having good SRH) and psychological subcomponent 7 (psc7: feeling useful). The unadjusted and adjusted ORs and their 95% CIs for the association of these remaining subcomponents with successful ageing are shown in Table 3. Participants having no heart disease (phc1) or having good SRH (psc4) at baseline were more likely to be successful agers after the 20-year follow-up even after the adjustments for age and gender. When these three subcomponents were included in a new model, the AUC for the ability to predict successful ageing was 0.673 and 0.823 in the unadjusted and adjusted analyses, respectively.

**Table 3:** The association of the remaining individual variables with successful ageing after the 20-year follow-up period

	Unadjusted			Adjusted^a^		
	OR (95% CI)	*P* value	AUC^b^	OR (95% CI)	*P* value	AUC^b^
Physical subcomponent 1:			0.673			0.823
Having no heart disease (ICD-10 I20‒25, I48‒49)	2.37 (1.48‒3.79)	< 0.001		1.85 (1.13‒3.03)	0.015	
Psychological subcomponent 4:						
Having good SRH	4.14 (1.66‒10.36)	0.002		2.73 (1.06‒7.03)	0.037	
Psychological subcomponent 7:						
Feeling useful	2.65 (1.63‒4.31)	< 0.001		1.64 (0.98‒2.75)	0.062	

OR = Odds Ratio

CI = Confidence Interval

AUC = Area Under the Curve

ICD-10 = 10th revision of the International Statistical Classification of Diseases and Related Health Problems

SRH = Self-Rated Health

^a^Adjusted for age and gender

^b^AUC for the model including the three subcomponents

### Re-examined successful agers

The number and proportion of the re-examined successful agers that met the criteria of the SA models at baseline and at re-examination are shown in Table 4. The number and proportion of the participants meeting the criteria of the models was lower at re-examination than at baseline. Still at re-examination, almost two-thirds of the re-examined successful agers, aged 84 to 96 years, were considered indeed as such by the SA model 3 which included the less demanding physical and psychological components. Also, 18, 27 and 38% of them met the criteria for models 1, 2 and 4, respectively.

**Table 4:** The number and proportion of the re-examined successful agers that met the criteria of the Successful Ageing models at baseline and at re-examination

*n* = 112	Baseline^a^		Re-examination	
Age, years	mean (SD) [range]			
	67.8 (2.5) [64‒77]		87.6 (2.5) [84‒96]	
	n (%)			
	Yes	No	Yes	No
Model 1 (PhC1 + PsC1 + SC)	50 (47)	56 (53)	20 (18)	92 (82)
Model 2 (PhC1 + PsC2 + SC)	62 (59)	43 (41)	30 (27)	82 (73)
Model 3 (PhC2 + PsC2 + SC)	81 (77)	24 (23)	73 (65)	39 (35)
Model 4 (PhC2 + PsC1 + SC)	66 (62)	40 (38)	43 (38)	69 (62)

PhC1 = Physical Component 1: Absence of all the diseases and independence in all five activities

PhC2 = Physical Component 2: Absence of dementia, less than three diseases, independent in three less demanding activities (move indoors, get in and out of bed, dress and undress)

PsC1 = Psychological Component 1: Absence of depression or depressive feelings, good self-rated health and leading a full life

PsC2 = Psychological Component 2: Absence of depression or depressive feelings and good self-rated health

SC = Social Component: Satisfaction in all three components of social relationships

SD = Standard Deviation

^a^At baseline, all the subcomponents of the SA models were not available for all the participants so the number of participants categorized by the SA models 1, 2, 3 and 4 was 106, 105, 105 and 106, respectively

## Discussion

According to the results of our study, meeting the criteria of any of the four SA models was associated with a higher probability of ageing successfully during the 20-year follow-up period both in the unadjusted, and adjusted analyses. The AUCs for the adjusted SA models were excellent.

However, after the backward stepwise logistic regression analysis, the three subcomponents found statistically significant in the unadjusted analyses were the absence of heart disease, having good SRH and feeling useful. Absence of heart disease and having good SRH were statistically significant also in the adjusted analyses. The AUC for the new model using these three subcomponents was as good as the AUCs of the SA models. In clinical practice, asking older people about their SRH and feeling of usefulness, and investigating whether they have heart disease could be of use in predicting ageing successfully, and also identifying those who have a lower probability of successful ageing in order to focus interventions on them.

The SA-models were constructed in line with the definition of Rowe and Kahn [2] and similarly to an earlier study on Finnish nonagenarians [5] including physical, psychological and social components. Model 1 is the most demanding model in regards to physical and psychological functioning while Model 3 can be considered as the least demanding model.

According to our results, successful ageing was possible also for persons with disease and disability, as it was not only the more strict SA Model 1 that was associated with a higher probability of successful ageing, but in fact, meeting the criteria of any of the models was associated with ageing successfully in the unadjusted, and the adjusted analyses. Our findings are in line with previous research stating that successful ageing is possible even with disease and disability [3], and seems to be a dynamic process of adapting to the accumulation of age, disease and functional decline [15].

The components and subcomponents used in our study mirror the study by Nosraty et al. (2019) [5]. Changes to the subcomponents were made only when a better substitute or no direct substitute was available. No major differences in the methods enables comparability to their work.

SRH is an individual’s own perception of their health and is influenced by their psychological characteristics, but even so, a predictor of survival [16], and institutionalization [17]. A study on an older Dutch population showed that while every new diagnosis gained was associated with poorer SRH, there seemed to be adaption to life with disease as the effect of a new diagnosis on SRH was dampened when the disease count increased [7]. This suggests that subjective SA can occur even in connection with multimorbidity [7], and thus supports the choice to include the SRH in the psychological component as it can also mirror the participants’ outlook on life and attitude towards hardship. In this study, the subcomponent of having a good SRH was associated with a higher probability, comparable to the multidimensional SA models, of successful ageing even after a 20-year follow-up period.

Life satisfaction and life meaning have earlier been shown to positively correlate with self-reported SA [18]. Gruenewald et al. (2007) found that older adults who more rarely reported feeling useful were more likely to experience disability and die during a 7-year follow-up period [19]. Also, Okamoto et al. (2007) reported that feeling useful was a better predictor of 6-year survival in older Japanese men than self-rated health (SRH) [20]. These earlier findings support our choice for the variables considered in the psychological component. In our study, feeling useful was associated with a higher probability of ageing successfully in the unadjusted analysis.

Our choice of diseases considered in this study were in line with the study by Nosraty et al. (2019) [5]. However, instead of self-report data used in the earlier study [5], we used the data gathered during the baseline examination, which included a review of the participants’ medical records and a physical examination, to determine whether the participants had any of the diseases considered in this study. This can be considered a strength to our study as self-report data can be inaccurate especially among older-aged women [21]. Also, when investigating the number and proportion of the re-examined successful agers meeting the criteria of the SA models at re-examination, great effort was made to include all the diagnoses acquired during the 20-year follow-up [10].

Having no heart disease at baseline was associated with a higher probability of successful ageing. This is probably due to the fact that the leading cause of death for people aged over 65 in Finland from 1997 to 2018 was cardiovascular diseases [22] and in this study, the original participants deceased before the re-examination were considered unsuccessful agers.

The three subcomponents (absence of heart disease, having good SRH and feeling useful) were probably found significant in this study because of the high cardiovascular mortality among Finnish older people [22], and the chosen definition of successful ageing. The ones able to live at home without need for daily care at the age of 84 years or older after the 20-year follow-up period are the ones who had not developed crucial disabilities at baseline or had the ability to cope with them.

The lay perspective of SA often includes social resources [23], and they are also often included in earlier [2], and also more contemporary multidimensional scientific SA-models [24]. A broader biomedical model including social aspects performed better at predicting quality of life in older adults than the mere biomedical model [25]. Having social support at time of adverse health events has been found to decrease the risk of institutionalization [9]. These earlier findings accentuate the need for social components to be included in SA models and support our inclusion of the SC in all the models. However, in our study, none of the subcomponents of the SC remained significant in the backward stepwise logistic regression analysis. An earlier study by Koutsogeorgou et al. (2015) investigating social capital and SRH in Finland, Poland and Spain found Finland to be a country of high social capita and the association between social capita and health was not significant in Finland as compared to Poland and Spain [26]. They argued that the relevance of social capital for health could be weaker in comprehensive welfare states, whereas social capital plays a more important role for SRH in countries with less comprehensive welfare states [26]. Whether including the SC in the SA models was an improvement on the quality of the measure cannot be said by this design. It could be of future interest to investigate whether the association of meeting the criteria of the SA models with successful ageing changes if the SC is omitted.

The strength of our study is the long follow-up period and meticulous review of the electronic patient record system for the dates on institutionalization and mortality and the presence of informal or formal care at time of the re-examination (exclusion criteria for successful agers). The finding that successful ageing could be predicted even 20 years beforehand gives ample opportunities for identifying also the older adults who currently are not on the trajectory for successful ageing and thus should be the targets for our interventions to improve their odds.

The weakness of our study are the elaborate and at least in the busy clinical setting unfeasible SA models that require a lot of information. That is why we also did the backward stepwise analysis to determine which of the subcomponents of the SA models were indeed significant, similarly as in our previous work when forming an easy-to-use index for predicting institutionalization and mortality among older people [27]. The backward stepwise analysis can be considered a strength to this study as it increases the feasibility of using our findings in a busy clinical setting. As we have suggested earlier [17, 28], including the older person’s subjective measure of health could be of use when formulating a personal care and rehabilitation plan for them.

Also a weakness to our study is the absence of some of the data on those successful agers that only gave their answers by mail. Because of that missing data, the number of participants that met the criteria of the SA models and their components at time of the re-examination is possible to report only for those successful agers that took part in the re-examination. When examining the number of participants that met the criteria of the SA models among the re-examined successful agers at re-examination, it is note-worthy that still 65% of them fulfilled the demands of the least demanding SA model 3 at the age of 84 years or older. They had no dementia, less than three diseases and were independent in three less demanding activities (PhC2). Also, 18, 27, and 38% of them met the criteria for models 1, 2, and 4, respectively. When investigating nonagenarians, Nosraty et al. (2019) found that 3.2, 9.2., 44.5, and 15.1% of the nonagenarians met the SA model criteria for models 1‒4, respectively [5]. In our study, the proportions were higher according to all of the models. It would be of interest to investigate whether these individuals continued to age successfully after the re-examination.

## Conclusions

Successful ageing models were associated with a higher probability of ageing successfully after a 20-year follow-up period. Using the backward stepwise analysis, we were able to identify the three most important subcomponents of the SA models: absence of heart disease, good SRH and feeling useful, and found the new model including these as good as in predicting successful ageing as the more elaborate SA models. Including these factors to the comprehensive geriatric assessment could help identify the possible successful agers, the ones who have the capacity to thrive and thus should be treated vigorously and not excluded from any treatments simply because of age. Also, identifying the older adults not on the trajectory to successful ageing could help us target interventions to improve their odds of ageing successfully.

### Electronic supplementary material

Below is the link to the electronic supplementary material.

Supplementary Material 1

Supplementary Material 2

## Data Availability

The datasets generated during and/or analyzed during the current study are available from the corresponding author on reasonable request.

## Abbreviations

ADL: Activities in daily living
AUC: Area under the curve
CI: Confidence interval
DSM-IV: Diagnostic and Statistical Manual of Mental Disorders
ICD-10: 10th revision of the International Statistical Classification of Diseases and Related Health Problems
OR: Odds ratio
PhC: Physical component
PsC: Psychological component
SA: Successful ageing
SC: Social Component
SD: Standard deviation
SRH: Self-rated health
