# Supplementary material for: Predicting successful ageing among older adults seems possible even as far as two decades ahead
Source: BMC Geriatr. 2024 Jun 1;24:481. doi: 10.1186/s12877-024-05109-8 (PMC11143575; doi:10.1186/s12877-024-05109-8)
Supplement: Supplementary file 1 — Supplementary Material 1 [file 12877_2024_5109_MOESM1_ESM.docx]

**Additional file 1.** The subcomponents of the components of the Successful Ageing models

| Component | Subcomponent | Explanation | ICD-10^a^ |
| --- | --- | --- | --- |
| Physical Component |  |  |  |
|  | phc1 | Not having heart disease, previously diagnosed or diagnosed at baseline examination | I20‒25, I48‒49 |
|  | phc2 | Not having cerebral vascular disease, previously diagnosed or diagnosed at baseline examination | I63‒64 |
|  | phc3 | Not having diabetes, previously diagnosed or diagnosed at baseline examination | E11 |
|  | phc4 | Not having arthritis, previously diagnosed or diagnosed at baseline examination | M05‒06, M10 |
|  | phc5 | Not having Parkinson’s disease, previously diagnosed or diagnosed at baseline examination | G20 |
|  | phc6 | Not having suffered a hip fracture | S72 |
|  | phc7 | Not having a previously diagnosed dementia | F00‒03, G30 |
|  | phc8 | Not having dementia according to the DSM-IV^b^ criteria at time of the baseline examination |  |
|  | phc9 | Not suffering from the effects of previous stroke at time of the baseline examination |  |
|  | phc10 | Able to move about indoors |  |
|  | phc11 | Able to get in and out of bed |  |
|  | phc12 | Able to dress and undress |  |
|  | phc13 | Able to walk a flight of stairs |  |
|  | phc14 | Able to walk 400 meters |  |
| Psychological Component |  |  |  |
|  | psc1 | Not having a previous diagnosis of depression | F32 |
|  | psc2 | Not having depression according to the DSM-IV^b^ criteria at time of the baseline examination |  |
|  | psc3 | Not having depressive feelings |  |
|  | psc4 | Having good self-rated health |  |
|  | psc5 | Being satisfied with life |  |
|  | psc6 | Looking hopefully into the future |  |
|  | psc7 | Feeling useful |  |
| Social Component |  |  |  |
|  | sc1 | Being satisfied with the relationship with one’s partner (or not having a partner) |  |
|  | sc2 | Being satisfied with the relationship with one’s children (or not having children) |  |
|  | sc3 | Being satisfied with the relationship with one’s friends |  |

phc = subcomponent of the Physical Component

psc = subcomponent of the Psychological Component

sc = subcomponent of the Social Component

Physical Component 1: Absence of all the diseases (subcomponents phc1‒9) and independence in all five activities (subcomponents phc10‒14)

Physical Component 2: Absence of dementia (subcomponents phc7‒8), less than three diseases (subcomponents phc1‒6 and phc9), independent in three less demanding activities (subcomponents phc10‒12)

Psychological Component 1: Absence of depression (subcomponents psc1‒2) or depressive feelings (subcomponent psc3), good self-rated health (subcomponent psc4) and leading a full life (subcomponent psc 5‒7)

Psychological Component 2: Absence of depression (subcomponents psc1‒2) or depressive feelings (subcomponent psc3) and good self-rated health (subcomponent psc4)

Social Component: Satisfaction in all three components of social relationships (subcomponents sc1‒3)

^a^10^th^ revision of the International Statistical Classification of Diseases and Related Health Problems

^b^Diagnostic and Statistical Manual of Mental Disorders
