# Supplementary material for: Predicting successful ageing among older adults seems possible even as far as two decades ahead
Source: BMC Geriatr. 2024 Jun 1;24:481. doi: 10.1186/s12877-024-05109-8 (PMC11143575; doi:10.1186/s12877-024-05109-8)
Supplement: Supplementary file 2 — Supplementary Material 2 [file 12877_2024_5109_MOESM2_ESM.docx]

**Additional file 2.** Number and proportion of participants who met the criteria for the physical, psychological and social components at baseline.

| *Component* | Number of participants meeting the criteria (percentage) | Number of participants not meeting the criteria (percentage) | Number of missing values |
| --- | --- | --- | --- |
| Physical component 1 (PhC1) | 610 (49) | 635 (51) | 15 |
| Physical component 2  (PhC2) | 1083 (88) | 154 (12) | 23 |
| Psychological component 1  (PsC1) | 552 (46) | 661 (54) | 47 |
| Psychological component 2  (PsC2) | 826 (69) | 368 (31) | 66 |
| Social component  (SC) | 1078 (87) | 160 (13) | 22 |
| *Subcomponent* | | | |
| phc1  Not having heart disease | 878 (70) | 382 (30) | 0 |
| phc2  Not having cerebral vascular disease | 1257 (100) | 3 (0) | 0 |
| phc3  Not having diabetes | 1117 (89) | 143 (11) | 0 |
| phc4  Not having arthritis | 1170 (93) | 90 (7) | 0 |
| phc5  Not having Parkinson’s disease | 1259 (100) | 1 (1) | 0 |
| phc6  Not having suffered a hip fracture | 1240 (98) | 20 (2) | 0 |
| phc7  Not having a previously diagnosed dementia | 1189 (94) | 71 (6) | 0 |
| phc8  Not having dementia at time of the baseline examination | 1140 (91) | 112 (9) | 8 |
| phc9  Not suffering from the effects of stroke | 1177 (94) | 72 (6) | 11 |
| phc10  Able to move about indoors | 1213 (97) | 44 (4) | 3 |
| phc11  Able to get in and out of bed | 1198 (96) | 46 (4) | 16 |
| phc12  Able to dress and undress | 1166 (93) | 91 (7) | 3 |
| phc13  Able to walk a flight of stairs | 1110 (89) | 143 (11) | 7 |
| phc14  Able to walk 400 meters | 1099 (88) | 156 (12) | 5 |
| psc1  Not having a previous diagnosis of depression | 1223 (97) | 37 (3) | 0 |
| psc2  Not having depression at time of the baseline examination | 966 (80) | 238 (20) | 56 |
| psc3  Not having depressive feelings | 1133 (92) | 93 (8) | 34 |
| psc4  Having good self-rated health | 1064 (85) | 181 (15) | 15 |
| psc5  Satisfied with life | 1201 (94) | 45 (4) | 14 |
| psc6  Looking hopefully into the future | 914 (75) | 312 (25) | 34 |
| psc7  Feeling useful | 841 (69) | 385 (31) | 34 |
| sc1  Satisfied with the relationship with partner | 1180 (95) | 64 (5) | 16 |
| sc2  Satisfied with the relationship with children | 1223 (98) | 20 (2) | 17 |
| sc3  Satisfied with the relationship with friends | 1152 (93) | 92 (7) | 16 |

Physical Component 1: Absence of all the diseases and independence in all five activities

Physical Component 2: Absence of dementia, less than three diseases, independent in three less demanding activities (move indoors, get in and out of bed, dress and undress)

Psychological Component 1: Absence of depression or depressive feelings, good self-rated health and leading a full life

Psychological Component 2: Absence of depression or depressive feelings and good self-rated health

Social Component: Satisfaction in all three components of social relationships
